# Fast food and sweet intake pattern is directly associated with the prevalence of asthma in a Qatari population

**DOI:** 10.1038/s41430-021-00959-6

**Published:** 2021-06-24

**Authors:** Zumin Shi, Tahra El-Obeid, Zainab Meftah, Amal Alawi, Suad Said, Vijay Ganji

**Affiliations:** Human Nutrition Department, College of Health Sciences, QU Health, Qatar University, P.O. Box 2713, Doha, Qatar

**Keywords:** Nutrition disorders, Risk factors

## Abstract

**Objective:**

The relationship between dietary patterns and the prevalence of asthma is not well understood. We aimed to investigate the association between dietary patterns and asthma in adults in Qatar.

**Methods:**

In this study, cross-sectional data from the Qatar Biobank were used (*n* = 986). Participants were Qatari or long-term Qatar residents aged ≥20 years old. A food frequency questionnaire was used to collect dietary intakes. Three dietary patterns were identified using factor analysis. Multivariable logistic regression was used to assess the association between dietary patterns scores and asthma.

**Results:**

Among 986 eligible participants, 6.6% (*n* = 65) reported that they were diagnosed with asthma. Three dietary patterns were identified. These were (1) “Traditional” (high intake of rice, chicken/meat/fish, and breads); (2) “Prudent” (high intake of fruits, vegetables, and fish); and (3) “Fast Food/Sweets” (high intake of desserts, fast food, and soft drinks). The fast food/sweet dietary pattern was associated with increased likelihood of having asthma [comparing high vs. low tertile, OR for asthma = 1.25; 95% CI (1.02–1.54); *p* = 0.035]. Traditional and Prudent dietary patterns were not associated with the prevalence of asthma.

**Conclusion:**

The fast food/sweet dietary pattern was directly associated with the prevalence of asthma among adults in Qatar. Reducing the fast foods and sugary-rich foods may be beneficial for respiratory health.

## Introduction

Based on the Global Burden of Disease Study, ~0.4 million people died from asthma in 2015 [1]. The risk of asthma increases by 50% in every 10 years of a person’s life [2]. In Qatar, the prevalence of asthma is 9% among adults [3]. The known risk factors of asthma are genetics, age, gender, and environmental factors [4]. Diet is considered as a modifiable risk factor for the progression of chronic diseases, such as respiratory disorders [5]. Recent evidence suggests that individual food and nutrients are associated with asthma [6–10]. Intakes of dietary antioxidants [5], fiber, fruit and vegetable [11], fish, and omega-3 fatty acids [12] have been shown to be inversely associated with asthma risk. This is more likely due to their anti-inflammatory effects. In contrast, high intake of fast foods [13] and sodium [11] is directly related to asthma.

During the past decade, there was an increased number of studies reported an association between food intake patterns and asthma [14–18]. However, findings from these studies are inconclusive with a substantial heterogeneities. Several studies have found that Mediterranean diet had a protective effect against asthma [14, 15, 17, 18]. On the other hand, Western dietary pattern with high intake of energy dense and processed foods had a direct association with asthma [19]. Some studies found no association between dietary patterns and prevalence of asthma [20, 21].

A limited number of studies have examined the association between diet and asthma in Qatar [22]. However, the association between diet and the prevalence of asthma has never been investigated in a national sample representing Qatar population. Therefore, the aim of the study was to investigate the association between dietary patterns and prevalence of asthma among Qatari adults.

## Materials and methods

### Study design and sample derivation

Since 2012 Qatar Biobank (QBB) has been collecting the demographic, health, and nutrition-related data prospectively from adults aged ≥18 years old who are Qatari nationals or those who have lived in the country ≥15 years. The details of the study design were published elsewhere [23]. A self-administered questionnaire was used to collect sociodemographic information, lifestyle factors, and dietary habits. Information on disease history and medication use were collected during a nurse interview. Participants were invited to have a health examination in the QBB facility at Hamad Medical City, Doha. Body weight, height, and blood pressure were measured by nurses. Blood samples (≈60 ml) were drawn and sent to Hamad Medical Corporation, Doha, Qatar for further processing.

In this current study, we used a random sample of 1000 participants from QBB database. The inclusion criteria were: aged ≥20 years old; answered the asthma-related question; and completed the food frequency questionnaire (FFQ). Of the 1000 participants, 14 were excluded due to the lack of data on asthma. Thus, the final study sample consisted of 986 participants. The QBB Survey was approved by the Institutional Review Board (IRB) from the Hamad Medical Corporation Ethics Committee. All participants gave written informed consent. The current analysis was approved under the IRB exempted category (Ex-2018-RES-ACC-0125-0069).

### Derivation of dietary patterns

Habitual food intake of participants was assessed using a qualitative FFQ. The FFQ was pre-tested for its internal validity before its use in the survey. Internal validity of the FFQ was tested by examining the frequency of consumption of broad categories of foods (meat, chicken, fish, salads, snacks, and fast/take away foods) in relation to the frequency of the consumption of the sum of the individual foods within the broad category using Spearman’s rank correlation test. The correlations (rho) ranged from moderate (0.3 for snacks intake) to high (0.74 for fish intake) (23). The FFQ included 102 food items consumed by Qatari population. Individual food consumption a year before survey was recoded into times consumed per week. In this analysis, based on the similarity of nutrition composition and cooking methods, food intakes were categorized into 38 food groups. These food groups were meat (beef and lamb), fish, chicken, chicken/meat fish mixed dish, grilled/fried/baked fish, eggs, white rice, biryani, zaata fatayer, Arabic/Iranian bread, brown bread, white bread, other breads, croissant, Asian noodles, soups/starters, salad and cooked vegetables, salad and raw vegetables, fresh fruit, fresh fruit juice, canned/dried fruit and dates, nuts, potato, lasagna, milk, cheese, yogurt, butter, breakfast cereal, milk added to cereal, coffee, tea, milk shakes, chocolate, desserts, ice creams, soft drinks, and fast foods. Factor analysis was used to construct dietary patterns. Varimax rotation method was used in the interpretation of the derived factors. We used Eigenvalues >1, Scree plot, and interpretability to determine the number of factors to be extracted. Dietary pattern scores were calculated for each individual based on pattern-specific factor loadings of each food group. The absolute value of a factor loading of >0.2 was regarded as the contributor for that dietary pattern. In the analysis, factor scores were used as continuous measure and categorical measure (tertiles: low, medium, and high).

### Determination of asthma cases

Asthma was self-reported by participants. Participants were asked whether they were diagnosed having asthma. Subjects who reported “yes” to the above question were considered having asthma.

### Confounding variables

In the study, we used age (continuous), gender, education (low: primary and secondary school; medium: technical or professional school; and high: university and postgraduate degree), leisure time physical activity level [metabolic equivalent of task, recoded as tertiles], smoking (non-smokers, ex-smokers, and current smokers), and body mass index (BMI) as confounding variables. Overweight and obesity were defined as a BMI of 25–29.9 kg/m and ≥30 kg/m^2^, respectively. Subjects with missing data for BMI were categorized as “not reported”.

### Statistical analysis

The data analysis was performed using the statistical software package Stata (version 15.1, College Station, TX, USA). Mean, standard deviation, and proportions were used to characterize the study sample. Factor analysis with principle component method was used to construct dietary patterns. Three criteria were used to determine the number of dietary factors. These were scree plot; Eigenvalues >1; and interpretability of the dietary factors. The associations between dietary pattern scores and the prevalence of asthma were analyzed using multivariate logistic regression. Three logistic regression models were developed. These were unadjusted, adjusted for age (continuous) and gender; and adjusted for age, gender, smoking, education, leisure time physical activity, and BMI (normal, overweight, obese, and not reported). We performed two separate analyses using dietary pattern scores as continuous measure and categorical measure (tertiles). The statistical significance was at *α* = 0.05.

## Results

Of 986 participants, 498 were men and 488 were women. The mean age of the study population was 39.5 years. The majority of participants were well educated (61.8% with high education and 27.7% with medium education) and non-smokers (68%). Out of 986, 65 individuals reported asthma, a prevalence of 6.6%. Although more reported asthma cases were among women than men (prevalence of 7.6% compared to 5.6%), there was no statistical significant difference between men and women (*p* = 0.22). The detailed characteristics of the study participants are reported in Table 1.

**Table 1:** Sample characteristics of study population (*n* = 986)^a^.

	Total	Men	Women	*p* value^b^
	*n* = 986	*n* = 498	*n* = 488	
Age, years	39.5 ± 11.8	39.1 ± 11.1	40.0 ± 12.5	0.21
Age categories				0.25
<40 years, *n* (%)	533 (54.1)	282 (56.6)	251 (51.4)	
40–60 years, *n* (%)	398 (40.4)	191 (38.4)	207 (42.4)	
>60 years, *n* (%)	55 (5.6)	25 (5)	30 (6.1)	
Education				<0.001
Low, *n* (%)	103 (10.5)	37 (7.4)	66 (13.5)	
Medium, *n* (%)	273 (27.7)	169 (34)	104 (21.3)	
High, *n* (%)	609 (61.8)	291 (58.6)	318 (65.2)	
Smoking status				<0.001
Non-smoker, *n* (%)	670 (68)	197 (39.6)	473 (96.9)	
Current smoker, *n* (%)	180 (18.3)	174 (34.9)	6 (1.2)	
Former smoker, *n* (%)	136 (13.8)	127 (25.5)	9 (1.8)	
Body mass index, kg/m^2^	28.9 ± 5.6	28.2 ± 4.7	29.5 ± 6.3	<0.001
Leisure time physical activity, MET hours/week	18.4 ± 38.5	21.8 ± 45	14.9 ± 30.1	0.004
Asthma prevalence, *n* (%)	65 (6.6)	28 (5.6)	37 (7.6)	0.22

^a^Data from Qatar Biobank. Values are mean ± standard deviation for continuous measures and *n* (%) for categorical measures.

^b^Significance between men and women. Unpaired *t*-test for continuous measures and Chi-squared test for categorical measures.

**Fig. 1:**
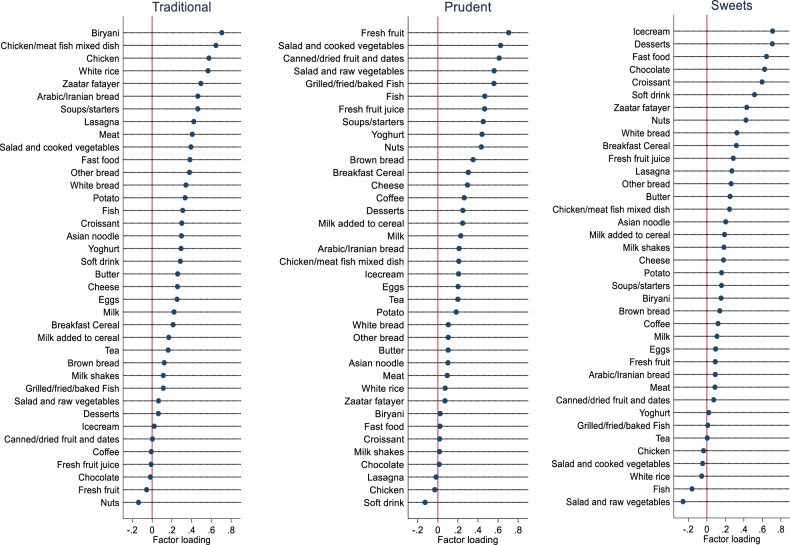
Factor loadings of three food intake patterns. Food intakes from 102-item qualitative food frequency questionnaire were categorized into 38 food groups. Factor analysis was performed to derive three food intake patterns (Traditional, Prudent, and Fast Food/Sweets) based on eigenvalues (>1.0), scree plot, and interpretability (*n* = 986). Data derived from Qatar Biobank.

We derived three dietary patterns using factor analysis. These were labelled as Traditional, Prudent, and Fast Food/Sweet dietary patterns. The “Traditional” dietary pattern had high factor loadings for biryani, rice, chicken/meat/fish mixed dish, chicken, white rice, zaatar fatayer, Arabic/Iranian bread, soups/starters, lasagna, meat, salad and cooked vegetables, fast food, white bread, potato, and fish. The “Prudent” dietary pattern had high factor loadings for fresh fruit, salad and cooked vegetables, canned/dried fruits and dates, salad and raw vegetables, grilled/fried/baked fish, fish, fresh fruit juices, soups/starters, yoghurt, nuts, brown bread, breakfast cereal, cheese, and coffee. The fast food/sweet dietary pattern had high factor loadings for ice-cream, desserts, fast food, chocolate, croissant, soft drink, nuts, and white bread. A detailed factor loadings of 38 food groups for three food intake patterns derived are presented in Fig. 1. A total variance in dietary intake explained by the three dietary patterns was 30.6% (Traditional, 18.2%; Prudent, 7%; and fast food/sweet, 5.4%).

The association between Traditional, Prudent, and fast food/sweet food intake pattern score as a continuous measure and the prevalence of asthma is shown in Table 2. There was a statistically significant, direct association between fast food/sweet dietary pattern scores (continuous) and the prevalence of asthma in unadjusted and age- and gender-adjusted model. After adjusting the analysis for age, gender, smoking, education, leisure time physical activity, and BMI, the association between fast food/sweet dietary pattern scores and the prevalence of asthma remained statistically significant (OR 1.26; *p* = 0.03).

**Table 2:** Association between food intake pattern scores as continuous variable and prevalence of asthma in Qatar population (*n* = 986)^a^.

	Traditional		Prudent		Fast food/sweet	
	OR (95% CI)	*p* value	OR (95% CI)	*p* value	OR (95% CI)	*p* value^b^
Unadjusted	0.94 (0.73–1.2)	0.61	0.93 (0.73–1.2)	0.59	1.22 (1–1.49)	0.049
Age- and gender-adjusted	0.96 (0.75–1.23)	0.75	0.89 (0.67–1.17)	0.4	1.25 (1.02–1.54)	0.03
Multivariate-adjusted^c^	0.95 (0.73–1.24)	0.72	0.9 (0.67–1.21)	0.47	1.26 (1.02–1.55)	0.03

^a^Data from Qatar Biobank. Values are odds ratios and 95% confidence intervals. Three dietary patterns were derived from factor analysis based on Scree plot, eigenvalues >1, and interpretability of dietary factors.

^b^Significance in logistic regression. Dietary pattern scores were used as continuous variable.

^c^Adjusted for age (continuous), gender, smoking, education, leisure time physical activity, and BMI (continuous).

The association between Traditional, Prudent, and fast food/sweet intake pattern score as a categorical measure with the prevalence of asthma is shown in Table 3. The fast food/sweet intake pattern was directly related to the prevalence of asthma (*p* = 0.046). In the fast food/sweet category, the likelihood of having asthma is two times higher in the third quartile compared to the first tertile dietary pattern score (OR, 2.04; 95% CI, 1.001–4.16). No association was observed between Traditional and Prudent dietary scores and the prevalence of asthma.

**Table 3:** Association between tertiles of food intake pattern scores and prevalence of asthma in Qatar population (*n* = 986)^a^.

Food intake pattern scores				
	Tertile 1	Tertile 2	Tertile 3	*p* value^b^
Traditional	≤−0.46	−0.45–0.22	>0.22	
* n*	328	329	329	
Cases	24	23	18	
Prevalence, %	7.3	7	5.5	
OR (95% CI)	1	0.83 (0.44–1.58)	0.72 (0.37–1.41)	0.34
Prudent	≤−0.48	−0.47–0.27	>0.27	
* n*	331	326	329	
Cases	21	21	23	
Prevalence, %	6.3	6.4	7	
OR (95% CI)	1	1.16 (0.58–2.32)	1.16 (0.56–2.38)	0.7
Sweets/fast food	≤−0.45	−0.44–0.14	>0.14	
* n*	327	331	328	
Cases	18	21	26	
Prevalence, %	5.5	6.3	7.9	
OR (95% CI)	1	1.39 (0.67–2.87)	2.04 (1.001–4.16)	0.046

^a^Data from Qatar Biobank. Values are odds ratios and 95% confidence intervals. Three dietary patterns were derived from factor analysis based on Scree plot, eigenvalues >1, and interpretability of dietary factors.

^b^Significance in multivariate logistic regression. Food pattern scores were used as categorical variable. Model was adjusted for age (continuous), gender, education, smoking, physical activity, and BMI (continuous).

## Discussion

In this study, we investigated the relation between dietary patterns and asthma using the data from QBB. To our knowledge, these are the first findings on the relationship between dietary patterns and the prevalence of asthma in a Qatar population. In this study, we found a significant direct association between consumption of fast food/sweet dietary pattern and the prevalence of asthma using factor analysis. The factor analysis is a widely used tool in studies investigating the relationship between food intake and health outcomes in epidemiological studies. Number of factors/dietary patterns extracted in this study are similar to the published studies using dietary intakes from FFQ. Dietary patterns reported in this study (Traditional, Prudent, and Fast Food/Sweets) are similar to other studies published from this region [24–27]. In Iranian population, Esmaillzadeh and Azadbakht [24] identified three dietary patterns. These were healthy (similar to Prudent), Western (similar to unhealthy), and Iranian (similar to Traditional) patterns. In Jordanian population, Tayyem et al. [25] reported three intake patterns, i.e., fast food (same as ours), Mediterranean (similar to Prudent), and high protein (similar to Traditional) dietary patterns. Further, in this current study, the three dietary patterns explained 30.6% of variance in food intake. For example, Ganji et al. [28] using the data from the National Health and Nutrition Examination Surveys on the US population reported a total of 27.5% variance in food intake from the three dietary patterns that they studied. In the Jordanian study, authors [25] reported that the variance explained by three dietary patterns was 35.4%. Thus, the total variance explained by three dietary patterns in this study by and large similar to the data published in the literature.

Direct comparisons between dietary patterns identified in our study and with other studies are difficult to perform due to the differences in food intakes, grouping of foods, cutoff eigenvalues used, and number of factors extracted. In this study, we found that frequent consumption of fast food/sweet dietary pattern which was heavily loaded with sugary food and fast food and this pattern was directly associated with asthma. This finding is inconsistent with published reports [19, 29, 30]. Frequent consumption of foods high in sugars could increase susceptibility to allergic airways inflammation, promote oxidative stress [31], induce obesity, and increase respiratory dysfunction (such as asthma symptoms) [32]. In addition, fast foods tend to be high in saturated fats, trans fats, and sodium. High levels of fat could suppress the immune system and cause atrophy of airways, induce obesity and arise oxidative stress and complications including asthma [33]. It is known that Qatar has a high prevalence of overweight/obesity and diabetes [34]. These findings emphasize the importance of promoting healthy eating.

We found no relationship between Traditional and Prudent dietary patterns and asthma prevalence. A study in France found that consumption of Western diet was associated with an increased risk of frequent asthma attacks [29]. Although a review of ten observational studies conducted among adults in North America, Europe, and Asia did not support an association between Western diet and asthma incidence or prevalence. However, it suggested a possible link between Western pattern and asthma morbidity [19]. We were unable to assess the relationship between dietary pattern and asthma morbidity as these data are not available in QBB. Similar to the Western food pattern, the association between Prudent pattern or Traditional pattern and lung function is also inconsistent. Although some studies showed that fruits and vegetables [6, 10, 35] and fish (Prudent diet) [8, 36] had a protective effect on asthma, several studies failed to prove that relation. Prudent dietary pattern which consists mainly of “fruits, vegetables, and fish”, is inversely associated with asthma [20, 29]. In a randomized clinical trial, consumption of diet low in fruits and vegetables has been shown to increase the risk of asthma exacerbation among asthmatic individuals [37]. A cohort study in China showed that Traditional dietary pattern was a risk factor for asthma [38]. Another study found that Traditional pattern had a detrimental impact on lung function [39]. However, several studies were consistent with our study and reported that there were no significant associations between Traditional food pattern and asthma [20, 39].

A strength of this study is the large sample size. This increases the precision of estimates. In the QBB survey, various demographic, health, and nutritional markers were collected from the participants. This allowed us to adjust regression models with various confounding variables. Because the data used in this study were taken from a random sample of Qatari population, results of this study can be applied to the Qatar population at large. Another strength is that the FFQ used in this study has covered a wide range of foods (102 items) commonly consumed in Qatar [40].

Because this study is based on a cross-sectional design, the cause and effect relationship should not be assumed. Another limitation of the study is the use of FFQ without portion sizes. The qualitative FFQ (i.e., without portion size) has been used to construct meaningful dietary patterns [40]. Moreover, the data on portion size add very little to the food intake variance and most of the variance is explained by frequency of consumption [41]. As with many dietary intake studies, dietary intake assessment with FFQ is also prone to measurement error due to subjects’ inability to recall foods consumed accurately [42]. Due to the lack of homogeneity between men and women in various demographic characteristics of the sample, the results of this study may not be extrapolated to the population at large. Finally, asthma was self-reported by the subjects. So, it is possible that the prevalence of asthma may have been under- or over-reported. We were unable to verify that the asthma cases were diagnosed by a physician. However, it is not known how this misreporting might have affected the results of this study.

In conclusion, fast food/sweet pattern was directly associated with the prevalence of asthma in adult Qatar population. Traditional and Prudent dietary patterns were not associated with asthma. Promoting food habits leading to the reduced consumption of foods high in sugar and fast foods may be beneficial in improving the lung health of Qatar population. Long-term studies are needed on the effects of fast food and sweet intake in the etiology and pathogenesis of asthma.
